# Supplementary material for: The Evolution and Characterization of the RNA Interference Pathways in Lophotrochozoa
Source: Genome Biol Evol. 2024 May 7;16(5):evae098. doi: 10.1093/gbe/evae098 (PMC11114477; doi:10.1093/gbe/evae098)
Supplement: evae098_Supplementary_Data [file evae098_supplementary_data.zip › Supplementary_materials.zip!/selected_miRNAs/NoGe_selected_miRNAs/known_miRNA_families_pdf_files/NMRB01003740.1_Notospermus_geniculatus_strain_Ushimado_scaffold3740,_whole_genome_shotgun_sequence_44407.pdf]

Provisional ID	:	NMRB01003740.1_Notospermus_geniculatus_strain_Ushimado_scaffold3740,_whole_genome_shotgun_sequence_44407
Score total	:	155551.3
Score for star read(s)	:	3.9
Score for read counts	:	155542.7
Score for mfe	:	1.7
Score for randfold	:	0
Score for cons. seed	:	3
Total read count	:	305102
Mature read count	:	303575
Loop read count	:	0
Star read count	:	1527

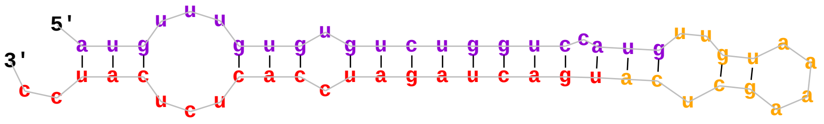

Genome coordinates: NMRB01003740.1 14646-14702

pre-miRNA sequence:
AUGUUUGUGUGUCUGGUCCAUGAUGUUUGUGUGUCUGGUCCAUGUUGUAAAAGCUCAUGACUAGAUCCACUCUCAUCC

Mature sequence: UGACUAGAUCCACUCUCAUCC

Aligned mature sequence of the *N. geniculatus* pre-miRNA with the top 5 hits from the BLAST search against the pre-miRNA mirGeneDB set:

```
                              ************* *******
Nge_Mir-279                   ugacuagauccacucucaucc-
Dpu-Mir-279-o6_3p             ugacuagauccacacucaucca
Dma-Mir-279-o6_3p             ugacuagauccacacucaucca
Bge-Mir-279-o36_3p            ugacuagauccacacucaucca
Isc-Mir-279_3p                ugacuagauccacacucaucca
```
